# Decision Making Within and Outside Standard Operating Procedures: Paradoxical Use of Operational Discretion in Firefighters

**DOI:** 10.1177/00187208211041860

**Published:** 2021-09-20

**Authors:** Philip C. Butler, Andy Bowers, Andrew P. Smith, Sabrina R. Cohen-Hatton, Robert C. Honey

**Affiliations:** 1 2112 Cardiff University, UK; 2 6697 University of Portsmouth, Hampshire, UK

**Keywords:** SOPs, acute stress, emergency services, operational discretion

## Abstract

**Objective:**

To understand how firefighters’ use of rules (i.e., standard operating procedures [SOPs]) and deliberative decision making (i.e., operational discretion [OD]) interacts with acute stress.

**Background:**

Current operational guidance for UK firefighters combines the provision of SOPs, for routine incidents, with the use of OD, under prescribed conditions (e.g., when there is a risk to human life). However, our understanding of the use of SOPs and OD is limited.

**Methods:**

Incident commanders (ICs; *n* = 43) responded to simulated emergency incidents, which either licensed the use of OD or required use of a SOP. Video footage of IC behavior was used to code their response as involving a SOP or OD, while levels of acute stress were assessed using a blood-based measure and self-report.

**Results:**

ICs were *less* likely to use OD selectively in the simulated emergency incident that licensed its use than in the one for which use of an SOP was appropriate; IC command level did not affect this pattern of results; and the incident that licensed OD resulted in more acute stress than the incident that required use of a SOP.

**Conclusion:**

SOPs and OD were not used in the manner prescribed by current operational guidance in simulated emergency incidents.

**Application:**

These results suggest that firefighter training in SOPs and OD should be augmented alongside personal resilience training, given the impact of stress on health and wellbeing, but also to improve the deployment of SOPs and OD under stress.

## Introduction

The economic impact of fire in the UK in 1 year alone was estimated to be £8.3B (Department for Communities and Local Government, 2011), but fire also has profound environmental and societal impacts. These impacts can be mitigated through the decisions made by first responders (e.g., firefighters). The routine nature of some emergencies means that decision making can be supported by explicit rules (i.e., standard operating procedures [SOPs]) or implicit learned rules (Epstein, 1994; Gigerenzer & Goldstein, 1996; Klein, 1993; Sloman, 1996). However, “unprecedented” emergencies (e.g., the Grenfell Tower fire) require a more flexible, deliberative approach where options are weighed against one another in terms of their potential costs and benefits (Kahneman, 2003; Neumann & Morgenstern, 1944). Within the UK fire and rescue service (FRS), firefighters have explicit rules (SOPs) for dealing with routine emergencies (e.g., a contained fire in a flat where there was no immediate danger to human life or property), and specified conditions that license departure from them and the use of operational discretion (OD; National Operational Guidance, 2018). The specified conditions that license such departure include “saving human life, taking decisive action to prevent an incident escalating, and incidents where taking no action may lead others to put themselves in danger*.*” This approach to how decisions are made balances the efficiency of rules with the flexibility afforded by the (conditional) use of deliberation to respond to a wide variety of emergencies. It forms part of the training and accreditation of UK firefighters, and specifically incident commanders who are responsible for directing the actions of firefighter crews at emergency incidents.

### Evidence From Laboratory Research

A paradox arises between the conditions under which OD is licensed and converging laboratory research about the conditions that influence the use automatic, rule-based processes rather than deliberative decision making. The conditions in which firefighters are licensed to depart from rules and to use OD (e.g., saving human life) are likely to generate acute stress (Lazarus & Folkman, 1984); acute stress can reduce the capacity for deliberative decision making and increase the reliance on rules in a variety of contexts (Porcelli & Delgado, 2009; Schwabe et al., 2012; Starcke & Brand, 2012; see also, Janis & Mann, 1977; Kassam et al., 2009; Peters et al., 2017; Porcelli & Delgado, 2017). For example, acute (extrinsic) stress exacerbates decision-making biases (in gambling tasks), which reflect the operation of automatic processes (Porcelli & Delgado, 2009; compare Kahneman & Tversky, 1979). Similarly, glucocorticoid and noradrenergic activation results in shift from goal-directed control of behavior to automatic, habitual control (Schwabe et al., 2012). Taken together, these results suggest that the very conditions under which firefighter guidance recommends the use of OD rather than the use of a SOP (i.e., when conditions are unprecedented and lives are at risk) might be expected to (indirectly) result in a greater tendency to use a SOP rather than OD.

### Naturalistic Decision Making

The paradox outlined above, however, is based upon an extrapolation from laboratory research, where the stressor can be the participants (usually undergraduate students) anticipating giving a public talk or having their hands placed in ice-cold water for 2 min (see Porcelli & Delgado, 2009). While these manipulations generate acute stress, they are unrepresentative of the conditions faced by firefighters who often work in challenging environments, which are characterized as time pressured, with high stakes and involving ill-structured problems (Orasanu & Connolly, 1993). Moreover, the nature of the firefighting role, the decisions that it entails (Klein, 1993), and indeed the individual characteristics of firefighters (Lazarus & Folkman, 1984; for reviews, see Mark & Smith, 2008; Salas et al., 1996) might mean that the results of laboratory research are of little relevance to firefighter decision making. The field of naturalistic decision making is concerned with just these issues, and studies within this field have revealed important insights into the nature of decision making in the world outside of the laboratory (see Zsambok & Klein, 2014). Our research is in that tradition. However, to the best of our knowledge, no study has assessed either (1) whether firefighters are more (or less) likely to depart from SOPs when the conditions are met to do so, and (2) whether or not those conditions are in fact perceived as stressful by firefighters (Lazarus & Folkman, 1984).

### Study and Predictions

To address these critical gaps in our knowledge, we examined the use of SOPs and OD by incident commanders (ICs). Incident commanders in the UK fire and rescue service have a multifaceted role. Briefly, they are expected to gather information that is relevant to the incident concerning resources and hazards in order to inform the selection of the appropriate course of action, and to communicate these actions to members of their crews, and other responding agencies. Here, ICs responded to two simulated incidents. (1) The Discretion scenario involved a group of children who had fallen into a sinkhole in a remote location, and licensed departure from the SOP (see Table 1) on the basis of, for example, saving human life. (2) The Control scenario involved a contained fire in a flat where there was no immediate danger to human life or property, which could be dealt with using the SOP (see Table 1). Video footage of the ICs was used to code their responses to the scenarios as involving the designated SOP or the use of OD, and we used a blood-based assessment of immune system function (Shelton-Rayner et al., 2010) and self-report to assess the levels of acute stress generated by the two incidents. On the basis of the laboratory research described above, we predicted that participants would be more reliant on the SOP and less likely to use OD in the Discretion than in the Control scenario, with the Discretion scenario generating higher levels of acute stress than the Control scenario. Finally, we examined the potential impact of command level (Klein, 1993; Klein et al., 1989) on the use of SOPs and OD and on acute stress, with the caveat that there were relatively few very senior ICs.

**Table 1:** Standard Operating Procedures and Examples of Operational Discretion

Scenario	Operational Responses
Discretion: Sinkhole rescue	**Standard operating procedure:** Enlist the support of specialist line rescue tactical advisers and teams to risk assess the situation and determine a plan to locate, rescue and recover the children to the surface. This would involve securing additional specialist equipment and techniques to safely lower Fire and Rescue Service (FRS) and medical personnel into the sinkhole to assess the situation and condition of the casualties and to carry out their work. **Operational discretion:** To save life, the committal of a firefighter down into the sinkhole using equipment designed to lower, but not raise, before the arrival of FRS specialist teams and equipment. **Operational discretion:** To save life, the committal of a Breathing Apparatus (BA) crew down into the sinkhole on two fully extended 10.5 m ladders tied together before the arrival of specialist FRS teams and equipment.
Control: High rise fire	**Standard operating procedure:** Following a risk assessment, establish a bridgehead, two floors below the fire floor, from which to launch a two-line attack. That is, two BA crews with hose lines, one to fight the fire in the flat, the other to protect their escape route (from the lobby) and enable their rescue if necessary. This would take a minimum of six personnel (a Bridgehead Officer, BA Entry Control Officer, and four BA wearers). **Operational discretion:** To prevent the situation from escalating from an established bridgehead, the committal of a single line attack (i.e., a single BA crew with a hose line), but without the required second BA crew to protect them.

## Method

### Participants

Forty-three incident commanders (42 male) volunteered from 15 UK Fire and Rescue Services (including three of the four UK nations) and provided informed consent for their participation in accordance with local ethical approval through the School of Psychology, Cardiff University. The use of a within-subjects design (with all participants receiving both scenarios) meant that the overall sample size was relatively large, while being determined by the availability and willingness of UK incident commanders to be involved in the research. The participants had a mean length of service of 22.84 years (range: 5.00–40.50 years), a mean length of experience in an incident commander role of 16.38 years (range: 2.67–30.00 years), and a mean length of service in current role (Level 2 or 3) of 4.05 years (range: .08–24 years). All participants were active incident commanders who were either at Level 2 (*n* = 32) or Level 3 (*n* = 11). Level 2 commanders are command and control middle managers at a tactical level, and Level 3 commanders operate at the tactical level at the scene of large and serious incidents. The participants wore standard issue fire service uniforms during the scenarios. The removal, storage, use, and disposal of blood samples were conducted in accordance with the Human Tissues Act 2004.

### Equipment

#### Questionnaires

Before undertaking the two simulated scenarios, all participants completed a suite of online questionnaires using Qualtrics software (Qualtrics, 2019). These included a stress-related questionnaire that combines the Smith Wellbeing questionnaire (SWELL), which focuses on occupational issues (Smith & Smith, 2017), with the Wellbeing Process Questionnaire (WPQ), which focuses on personality characteristics (Williams et al., 2017). Here, our main interest was in the level of acute stress during the two scenarios, but we also examined whether there was any relationship between chronic stress, as measured in the questionnaire, and our two measures of acute stress (LCC and self-reported stress); as we will show, there was not. Participants also completed a questionnaire to capture details of their operational experience across all levels of command they had practiced.

#### Simulation suite and apparatus

The simulations were conducted in a purpose-built incident command simulation suite at the Headquarters of Hampshire Fire and Rescue Service. The simulation suite consisted of a series of training rooms and a control room housing the equipment required to control the course of the simulated events: computers, audio and visual monitors, and communications equipment. During a simulation, the control room contained the simulation director, the radio communications role-player, and an XVR-trained technician to manipulate images of the incident. The moving images that represented the scene of the incident were displayed in a training room in which the simulated incident took place. These images were created and generated using XVR software. This room also acted as a holding area for all other role-players. Further details concerning the simulation suite and apparatus can be requested from the authors.

A large training room (H × L × W: 2.5 m × 10 m × 6 m) housed the mock command unit and a large monitor used to display a digital film of the changing situation at the scene. GoPro cameras were used to capture activity within this room. A digital clock, placed in the field of view of one of these cameras, enabled key events to be timed. Handheld radios were used for mobilizing control center and incident ground radio communications. A data projector was used to display command support software, such as a decision log and location information, maps, and images. Each simulation involved several generic role-players such as command unit officers, police and ambulance officers, along with role-players who were specific to the scenario (e.g., relatives of those involved, a line rescue tactical advisor, and an aerial ladder platform Crew manager). The command unit officers were trained staff who performed the role at real incidents. They were briefed to support the participants as they would commanders at a real incident, and provided with copies of the prescribed radio messages from the mobilizing control center and incident ground.

A smaller room (H × L × W: 2.5 m × 6 m × 4 m) was used to take blood samples before and after both scenarios, and to attach a chest-mounted GoPro camera to capture their conversations and verbalized thoughts. The blood samples were used to provide an assessment of the impact of the two scenarios on a marker of immune system function. Briefly, leukocytes are white blood cells that are involved in the immune system’s first response to threat of ill health caused by foreign bodies or stress. There are different types of white blood cells, with neutrophils representing the majority. One way neutrophils respond to stress is to release reactive oxygen species (ROS) and neutrophils circulating in the blood that have responded to one threat will have a reduced capacity to generate ROS to challenge another. Leukocyte Coping Capacity (LCC) is a measure of the ability of leukocyte (mainly neutrophils) to produce ROS in response to a chemical attack of phorbol myristate acetate (PMA; see Shelton-Rayner et al., 2010). The greater the level of neutrophil reactivity, the greater the ability to cope with stress. This measurement can be considered to represent an individual’s level of resilience to stress. For this study, LCC was measured using a test kit produced by Oxford MediStress Ltd (Oxford, UK), which includes a luminometer, heating block, pipette, buffer solution, and PMA reagent. For each of the four samples per individual, a trained researcher (PCB and AB) used a disposable blood lancet on a finger to generate a pinprick (10 micro-liters) of blood that was drawn off using a pipette. The blood was transferred to a glass luminometer tube held in a heating block at body temperature (37⁰C) containing the PMA reagent mixed with a buffer solution. After 10 min, the sample was tested by placing the glass tube in a luminometer and a reading of reactivity taken in Relative Light Units (RLU). Lower scores are associated with recent exposure to a stressor, and a reduced potential to cope with future stressors. In fact, the LLC scored were expressed as a ratio: LLC score after the scenario, relative to the sum of this score, and the LCC score before the scenario. Using this measure, scores below .50 indicate that the LCC score is lower after the scenario than before it. The LCC scores were complemented by self-report measures during the two scenarios. These were taken at four time points, approximately: 5, 12, 20, and 25 min after the start of each scenario. Participants verbally rated on a scale of 1 (“feeling no pressure”) to 10 (“unable to cope with the pressure”) how they were coping, when this information was requested by the Quality Assurance Officer (participants also wore heart-rate monitors, but these proved to be unreliable in approximately one-third of participants).

A final training room (H × L × W: 2.5 m × 6 m × 4 m) was used to debrief participants and to complete a semistructured interview after both simulations, which was cued by the presentation of a video of them completing the scenario. During the interview (mean duration = 1 hr, 8 min, and 39 s; range: 25 min and 22 s–1 hr, 55 min, and 3 s), participants were asked to recall their thoughts about their decision making at various points during the simulations, their stress levels, and the application (or not) of OD (see Appendix 1 for the questions). The answers provided during this interview were used to *inter alia* confirm the observed use of SOPs and OD from the recordings of the scenarios.

### Procedure

Participants were tested between August and November 2019, and received one scenario in the morning and the second in the afternoon. In between the two scenarios, participants had lunch. Approximately half of the participants (21) received the Discretion scenario in the morning and the Control scenario in the afternoon, and the remainder (22) received the reverse arrangement. Immediately before and after each scenario, a blood sample was taken from one of the participant’s fingers (and LCC was assessed). Participants were then taken to the room in which the scenarios were delivered. Before entering the room, the Quality Assurance Officer role-player gave the participants a general briefing on the time of year, day and the climatic conditions. They also read out a mobilizing message from the mobilizing control center that outlined basic information about the incident. The participants were given an opportunity to ask questions of the mobilizing control center, as would be the case at real incident.

#### Scenario generation

The two scenarios were designed and developed by two researchers (PCB and AB) who are recently retired, experienced incident commanders (advanced level commanders). The Discretion scenario was designed to replicate circumstances that licensed the application of OD, and the use professional judgment to make decisions (summarized in Table 1). This simulation involved five young children who had fallen down a deep sinkhole in a remote location, and included cues that related to each of the outcomes that justified (according to National Operational Guidance, 2018) the application of OD: saving human life, taking decisive action to prevent an incident escalating, and where inaction may lead others to put themselves in danger. The SOP in this case is to enlist the support of specialist line rescue tactical advisers and teams to risk assess the situation and determine a plan to locate, rescue, and recover them to the surface. This would involve securing additional specialist equipment and techniques to safely lower FRS and medical personnel into the sinkhole to assess the situation and condition of the casualties and to carry out their work. However, embedded within Discretion scenario were components that should have resulted in the use of OD. The Control simulation involved a fire in a high-rise block of residential flats, and included cues that informed the incident commander that the risks to life and property were low. As a result, the simulated incident could be successfully resolved, with minimal risk to firefighters and the public, by using the familiar SOP based on the service’s generic risk assessment for firefighting in high rise buildings (Chief Fire and Rescue Service Advisor, 2014) and national operational guidance (National Operational Guidance, 2019a): Following a risk assessment, establish a bridgehead from which to launch a two-line attack. That is, two breathing apparatus crews with hose lines, one to fight the fire in the flat, and the other to protect their escape route (from the lobby) and enable their rescue if necessary. This would take a minimum of six personnel (a Bridgehead Officer, BA Entry Control Officer and four BA wearers). There was no basis upon which to move beyond this SOP to resolve the incident.

#### Video scoring

During both scenarios, the ICs responded to the unfolding incident, complete with scheduled injects, in the way that they would a real incident: requesting information about resources and hazards, formulating plans, and directing the actions of their crew members. The responses of ICs to each scenario were video-recorded and later scored as either using the requisite SOP (Table 1) or departing from it and using OD. PCB scored videos from all ICs on two separate occasions to ensure the accuracy of the coding, and a subset of the videos were also scored by RCH to confirm the reliability of the categorical coding (interrater agreement = 100%). The semistructured interviews (see Appendix 1 for the Discretion scenario interview) provided another basis upon which to confirm that a SOP or OD had been applied, but the content of these interviews was not subject to any further formal analysis here.

## Results

### The Use of SOPs and OD

**Figure 1:**
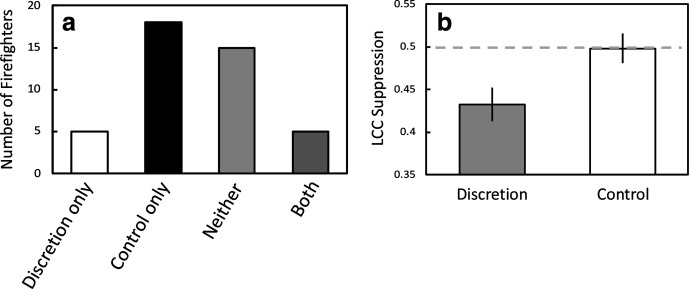
Results: Panel a shows the numbers of firefighters who exercised operational discretion in the two scenarios (Discretion and Control). Firefighters were classified as using operational discretion in: only the Discretion scenario; in only the Control scenario; in neither scenario; or in both scenarios. Panel b shows the mean suppression in LCC score (±SEM), relative to baseline, after participation in the two scenarios. LCC = Leukocyte Coping Capacity.

The overall results from the study were clear and are depicted in Figure 1a. Incident commanders were *less likely* to depart from using the SOP in the Discretion scenario, where such departures were licensed by the conditions, than in the Control scenario, where such departures were not licensed; examples of the use of OD in the two scenarios can be found in Table 1. Thus, only five used OD exclusively in the Discretion scenario, and a significantly greater number (18) used discretion exclusively in the Control scenario (binomial test, *p* = .01); five used OD in both scenarios and 15 did not use it in either (binomial test; *p* < .05). That is, there were more participants who did not use OD at all than participants who used OD in both scenarios. McNemar’s test confirmed that the proportions of the four types of response (represented by the four bars) differed (χ2 **=** 6.26, *p* = .01, OR = .28). Finally, the durations of the Discretion scenario (mean = 32.25 min; SEM = .45) did not differ significantly from those of the Control scenario (mean = 31.53 min, SEM = .66; *t*(42) = 1.05, *p* = .30, d = .158).

### Levels of Acute Stress in the Two Scenarios

The Discretion scenario resulted in more acute stress than the Control scenario, using both the blood-based assessment of leukocyte function (Figure 1b) and self-reported stress. Panel B shows the mean suppression in LCC after both scenarios: LCC score after the scenario/(LCC score after scenario + before scenario). As already noted, scores below .50 indicate a suppression in the LCC score after the scenario, with the degree of suppression indicating the capacity to cope with further stressors. The broken gray line indicates no suppression in the LCC score as a consequence of participation in the scenario. There was more suppression in LCC scores after the Discretion scenario than after the Control scenario (*t*(42) = 2.206, *p* < .05, d = .337); and one sample *t*-tests confirmed that the scores for the Discretion scenario were below .50 (*t*(42) = −3.391, *p* < .005, d = .51), whereas those for the Control scenario were not (*t*(42) = −.125, *p* = .902, d = .02).

The self-reported stress scores (minimum = 0 and maximum = 10) increased across both scenarios (Table 2). The analysis of variance (ANOVA) revealed no main effect of scenario, *F*(1, 42) = 1.473, *p* = .232, np^2^ = .034, a main effect of test (1-4), *F*(3, 126) =101.686, *p* < .001, np^2^ = .708, and an interaction between these factors, *F*(3, 126) = 2.671, *p* = .05, np^2^ = .060; with the scores for the Discretion scenario being higher than the Control scenario on test 2 (*t*(42) = 2.14, *p* < .05, d = .32). There was a *negative correlation* between the final self-reported stress score (high scores = more stress) from the Discretion scenario and the raw LCC scores (low scores = less residual capacity to cope with stress) taken after the scenario, using both Pearson’s (*r*_p_) and Spearman’s (*r*_s_) correlations (*r*_p_ = –.446, *p* < .005; *r*_s_ = –.439, *p* < .005), but there was no correlation for the corresponding scores for the Control scenario (*r*_p_ = −.036, *p* = .82; *r*_s_ = .051, *p* = .74). Given the fact that the Control scenario did not result in a suppression of LCC scores, the latter observation is not particularly surprising.

**Table 2:** Mean (+SEM) Self-Reported Stress at Four Successive Timepoints During the Two Scenarios

Timepoint	1	2	3	4
Discretion scenario	3.81 (0.24)	5.30 (0.24)	5.55 (0.22)	6.58 (0.20)
Control scenario	3.86 (0.26)	4.79 (0.25)	5.44 (0.25)	6.16 (0.22)

The chronic stress scores taken from the questionnaire (mean = 6.18, SEM = .29) did not correlate with the measures of acute stress during either scenario: LCC ratios from the Discretion scenario (*r*_p_ = −.08, *p* = .57; see Figure 1b for mean) or from the Control scenario (*r*_p_ = .06, *p* = .72; see Figure 1b for mean), or with self-reported stress scores on test 2 in which the Discretion and Control scenarios differed (*r*_p_ = .18, *p* = .25 and *r*_p_ = .25, *p* = .11, respectively).

### The Impact of Command Level

Of the 43 incident commanders, 32 were intermediate level and 11 were advanced level. The proportions of participants at the two levels who only used OD in either the Discretion scenario or Control scenario was consistent with the overall pattern of results depicted in Figure 1a: Intermediate level (4 versus 14) and Advanced level (1 versus 4). These proportions did not differ using a Fisher’s exact probability test (*p* > .05). However, there was some indication that the proportions that used OD in neither or both scenarios differed between the levels: Intermediate level (neither = 13 versus both = 1) and Advanced level (neither = 2 versus both = 4; *p* < .05). This difference, albeit with a very small number of advanced level incident commanders, suggests that a general reluctance to use OD was more evident in the intermediate level commanders than the advanced level commanders. A secondary analysis of the LCC suppression scores for the two scenarios, including the two command levels, revealed a similar pattern to that depicted in Figure 1b: intermediate = .44 (Discretion; SEM = .023) and .49 (Control; SEM = .016); and advanced = .40 (Discretion; SEM = .037) and .51 (Control; SEM = .051). The ANOVA revealed no effect of command level, *F*(1, 41) = .181, *p* > .67, np^2^ = .004, an effect of scenario, *F*(1, 41) = 5.59, *p* = .02, np^2^ = .12, and no interaction between these factors, *F*(1, 41) = .83, *p* > .36, np^2^ = .02.

## Discussion

Decisions made by firefighters can mitigate the economic, environmental, and social impacts of emergency incidents. Guidance given to firefighters in the UK Fire and Rescue Service recognizes two approaches to decision making: with the recommendation that responses to routine emergency incidents are based on rules (i.e., SOPs) and “unprecedented” incidents licensing the use of a more flexible, deliberative approach (i.e., OD; National Operational Guidance, 2019b). The recognition of these two processes is echoed in psychological theory, where the use of rules (Epstein, 1994; Gigerenzer & Goldstein, 1996; Klein, 1993; Sloman, 1996) is distinguished from a deliberative approach involving a cost–benefit analysis (Kahneman, 2003; Neumann & Morgenstern, 1944). Our research concerned the deployment of SOPs and use of OD in experienced firefighters. We used two scenarios: the Discretion scenario licensed the use of OD (e.g., on the basis of saving human life) and the Control scenario did not. The use of OD was neither random (equally evident in both scenarios) nor was it consistently used by different incident commanders (either always using it or never doing so). In fact, only five of the 43 firefighters used OD in a scenario-appropriate manner.

If we first consider the Control scenario alone: a fire in a flat in which there is no danger to human life. This is a relatively routine incident, for which the SOP is well established (Table 1), and participants were informed that there was no risk to human life; yet over half of the participants (23) used OD without justification for doing so. This observation is, in and of itself, important. The Discretion scenario was less routine, and there was a clear risk to human life involving the children who had fallen into a sinkhole. Taking this scenario alone, only 10 of the 43 participants used OD. Now taking the two scenarios together, there were more ICs who used OD in the Control scenario and not in the Discretion scenario than ICs who used OD in a context-appropriate manner. Whether the results of the scenarios are taken separately or together, they have important implications and prompt two questions: Why was OD used when an entirely appropriate SOP was available? Why when the conditions licensed OD was it not used? One possibility is informed by the fact that the Discretion scenario generated greater acute stress than the Control scenario, as measured by both immune function and self-report.

The results of laboratory studies show that extrinsic stress can result in a reliance on rules rather than deliberation (Kassam et al., 2009; Starcke & Brand, 2012; see also, Janis & Mann, 1977; Peters et al., 2017). By the same token, the fact that the Discretion scenario generated more stress than the Control scenario might have resulted in a greater reliance on SOPs than OD. But how is acute stress generated and how does it impact decision making? One influential class of psychobiological accounts assumes that acute stress is generated when the perceived demands of the situation are judged to be beyond the personal and environmental resources that are available to address those demands (see Lazarus & Folkman, 1984; Salas et al., 1996; for a review, see Mark & Smith, 2008). To the extent that the Discretion scenario involved such a mismatch, including the grounds for the use of OD, then it would be expected to generate acute stress. There are a variety of plausible mechanisms by which acute stress—generated in this way—could affect the use of SOPs and OD. For example, it could limit attentional resources and thereby constrain either (1) the capacity for the deliberative processes upon which OD relies (e.g., Combs & Taylor, 1952; Easterbrook, 1959), or (2) the requisite situational awareness (Endsley, 1995). In the next paragraphs, we explore the utility and limitations of the approach employed here, and the implications of our results for firefighter training and decision making.

### Limitations

The use of simulated emergencies enables levels of reproducibility and experimental control that would be impossible in real emergencies: in particular, incidents requiring the use of OD are relatively rare and the assessment of acute stress would be intrusive. However, simulations provide an incomplete representation of the variety and impacts of real emergency incidents on firefighter decision making. For example, while the two simulated scenarios employed here had the predicted effects on measures of acute stress, they are unlikely to generate the levels of acute stress experienced during real incidents. The study of complementary real-world incidents could clearly provide important converging evidence for conclusions based on those from simulations. It would also be beneficial to replicate the results reported here in a broader range of scenarios, but there are obvious constraints on the availability of our participants (i.e., incident commanders) to undertake research studies. Nevertheless, the overall similarity between decision-making processes observed in real emergency incidents (Cohen-Hatton et al., 2015) and a range of simulated ones (Cohen-Hatton & Honey, 2015) suggest that our results are very likely to generalize to real emergency incidents. Finally, it is possible that our two scenarios have independent effects on stress and the use of OD. However, this possibility leaves one without a ready explanation for why OD was less likely to be used selectively in the scenario in which it is licensed than the scenario in which it was not.

### Summary and Implications

The UK fire and rescue service guidance for operational decision making balances the efficiency of rules (i.e., SOPs) with the flexibility afforded by the (conditional) use of deliberation to respond to a wide variety of emergencies (i.e., OD). Our primary finding suggests that this balance is not reflected in operational decision making: OD was more likely to be deployed when it was not licensed (in the Control scenario) than when it was licensed (in the Discretion scenario). Taken in isolation, these results can be taken to suggest a need to reinforce operational guidance and training. However, our secondary observation that the two scenarios were associated with different levels of acute stress suggests that this approach might be ineffective: to the extent that incidents licensing the use of OD are likely to generate greater acute stress and this will affect the use of SOPs and OD. If one accepts the proposition that the use of OD and deliberation should be licensed under unprecedented conditions (e.g., the Grenfell Tower fire), then our results suggest a need for training to focus on generating effective decision making under stress, and specifically training to enhance personal resilience to mitigate the impact of acute stress on decision making (see Driskell et al., 2001; Saunders et al., 1996). The clear prediction is that such training would increase the use of OD when it is required. The results of a recent survey of training provided by UK fire and rescue services are illuminating in this respect: all of the fire and rescue services that responded (27; approximately half of the UK fire and rescue services) delivered training in decision making (25 involving both theoretical and practical components), while relatively few (14) provided training in any form of personal resilience (with only nine providing practical training; Butler et al., 2020; see also Sawhney et al., 2018). There are clear grounds to augment the training given to first responders in personal resilience, directed at mitigating the effects of acute stress, and to engender a culture in which different facets of incident command, including the effective use of OD, are integrated and supported.

## Key Points

- Firefighters receive guidance about when to use standard operating procedures (SOPs) and when operational discretion (OD) is licensed (e.g., on the grounds of saving human life). Here, firefighters responded to simulated scenarios that either required the use of a SOP or licensed the use of OD.
- OD was less likely to be used under circumstances in which it was licensed than when it was not, and the scenario that licensed the use of OD generated more acute stress than the scenario that required use of an SOP.
- These results provide an impetus for training that integrates consideration of the use of SOPs and OD alongside personal resilience (i.e., to reduce the impact of acute stressors in operational contexts).

## Appendix 1

### Interview questions for discretion scenario

#### Decision Point: Taking over/not taking over command

1. What was the rationale for your decision?
2. When you took over/did not take over command what did you understand about the incident?
  - The situation?
    - Have you had any command experience of this type of incident before?
  - The resources?
    - What was your rationale for increasing the resources (or did not)?
  - The hazards and risks?
    - What did perceive as the greatest hazards?
  - What were your information gaps?
    - Did you appreciate who was available and their skills and knowledge?
    - Did you fully trust the people involved?
    - Did you appreciate the capabilities of available appliances and equipment?
    - Were you aware of how much time had passed at this point?

#### Decision Point: Initial Plan

1. What was your plan at this stage?
  - How did you determine your objectives?
  - How did you determine your priorities?
  - What sources of knowledge were you relying on to determine your plan?
  - Were you relying on any SOPs, and if so, which ones?

#### Decision Point: Use of Initial Incident Commander

1. How did you utilize the initial incident commander?
  - What was the benefit of using them in this way (or not using them)?
  - What did you hope to achieve by using them this way?
  - Have you used them in this way before?

#### Decision Point: Need for additional resources

1. Why did you make up/Did you consider making up?
  - How did you/would you intend to use the resources for?
  - What influenced/would have influenced the number and type of appliances?
  - What cues did/would you use to decide the scale of the make up?

#### Decision Point: Need to provide incident update

1. Why did you send an informative message at this time/Did you consider sending an informative message?
  - What cues did/would you use to know when to send an informative?
  - What sources of knowledge did/would you use to determine the message content?
  - What rules did/would you follow?

#### Decision Point: Use of the parents

1. How did you handle and use the parents?
  - What was the rationale for your decision?
  - What were you uncertain about?
  - How did you come to that decision?
  - What was the benefit of your actions in relation to them?
  - What did you hope to achieve by those actions?
  - Have you done this with parents before and were the circumstances similar?

#### Decision Point: Use of the line rescue tactical adviser

1. How did you utilize the tactical adviser?
  - What was the rationale for your decision?
  - What were you uncertain about?
  - What was the benefit of using them in this way (or not using them)?
  - What did you hope to achieve by using them this way?
  - Have you used them in this way before?
  - What sources of knowledge did/would you use?
  - What were the rules/SOPs you were following?

#### Decision Point: Use of the police resources

1. How did you utilize the Police?
  - What was the rationale for your decision?
  - What were you uncertain about?
  - What was the benefit of using them in this way (or not using them)?
  - What did you hope to achieve by using them this way?
  - Have you used them in this way before?
  - What sources of knowledge did/would you use?
  - What were the rules/SOPs you were following?

#### Decision Point: Use of the HART resources

1. How did you utilize HART?
  - What was the rationale for your decision?
  - What were you uncertain about?
  - What was the benefit of using them in this way (or not using them)?
  - What did you hope to achieve by using them this way?
  - Have you used them in this way before?
  - What sources of knowledge did/would you use?
  - What were the rules/SOPs you were following?

#### Decision Point: Expansion and/or adaption of initial plan

1. What was your plan at this stage?
  - How do you feel the incident has developed?
  - How did you determine your objectives?
  - How did you determine your priorities?
  - What information has influenced the adaptation of your plan?
  - What were you uncertain about?
    - Did you appreciate who was available and their skills and knowledge?
    - Did you fully trust the people involved?
    - Did you appreciate the capabilities of available appliances and equipment?
    - Were you aware of how much time had passed at this point?
  - Were there any specific cues?
  - How do you feel the risks have changed?
  - What sources of knowledge were you relying on to do this?
  - Were you relying on any SOPs, and if so, which ones?

#### Decision Point: Use of tree surgeon parent

1. Why did you handle the tree surgeon parent in that way?
  - What was the rationale for your decision?
  - What were you uncertain about?
  - What was the benefit of your actions in relation to them?
  - What did you hope to achieve by those actions?
  - Have you done this before and were the circumstances similar?
  - Did you consider their expertise when making this decision?

#### Decision Point: Response to the stress question:

1. How were you feeling at this point?
2. What were you uncertain about?
3. What cues influenced your level of stress at this time?
4. What made it increase/decrease from before?
5. How comfortable at this point are you with your decisions?
6. How were you managing your stress?
7. Were you relying on your training?
8. Was that level of stress affecting your ability to command, and if so, how?
9. Did that level of stress affect your behavior, or other aspects of your performance?

#### Decision Point: Applies (or omits to apply) operational discretion appropriately or inappropriately

1. Were you aware of going outside of standard operational procedures?
2. What were you uncertain about?
3. What influenced your decision to do that/not do that?
4. What cues did you use?
5. What sources of knowledge were you relying on?
6. What is the procedure you should have followed?
7. Why did you stick to using SOPs?
8. Does your FRS have an Operational Discretion SOP?
9. Why did you not follow it?
10. Were there any organizational or cultural matters that influenced your approach?
